# Association between Glaucoma and Obstructive Sleep Apnea Syndrome: A Meta-Analysis and Systematic Review

**DOI:** 10.1371/journal.pone.0115625

**Published:** 2015-02-23

**Authors:** Yuhua Shi, Panpan Liu, Jian Guan, Yan Lu, Kaiming Su

**Affiliations:** 1 Department of Ophthalmology, Jinling Hospital, School of Medicine, Nanjing University, 305 East Zhongshan Road, Nanjing, China; 2 Department of Otolaryngology, Shanghai Jiao Tong University Affiliated Sixth People's Hospital, Otolaryngology Institute of Shanghai Jiao Tong University, Shanghai, China; Weill Cornell Medical College in Qatar, QATAR

## Abstract

**Background:**

Obstructive sleep apnea syndrome (OSAS) is a common disease that increases the risk of diabetes, heart disease, and stroke. However, studies of an association between OSAS and glaucoma neuropathy have reported controversial findings.

**Objective:**

The main purpose of this study was to evaluate whether a significant association exists between OSAS and glaucoma by performing a meta-analysis of previous studies.

**Methods:**

A comprehensive literature search using the PubMed and Embase databases was performed to identify cross-sectional, case-control, and cohort studies related to the topic. We estimated a pooled odds ratio (OR) for the association between OSAS and glaucoma, by a fixed- or random-effects model.

**Results:**

In total, 16 studies with 2,278,832 participants met the inclusion criteria: one retrospective cohort study reported an adjusted hazard ratio of glaucoma of 1.67 (95% CI = 1.30–2.17). Using a fixed-effects model, the pooled OR of six case-control studies was 1.96 (95% CI = 1.37 2.80). A significant association was also identified in a meta-analysis of nine cross-sectional studies using a random-effects model, which showed a pooled OR of 1.41 (95% CI = 1.11 1.79). However, the reported pooled estimates for case control studies and cross-sectional studies were based on unadjusted ORs.

**Conclusions:**

Our results suggest that OSAS is associated with the prevalence of glaucoma. However, this result was based only on unadjusted estimates. Prospective cohort studies designed to take into consideration potential confounders, or examination of data from interventional trials to determine whether a reduction in OSAS status is associated with a reduced incidence of glaucoma, are needed to clarify whether OSAS is an independent risk factor for glaucoma.

## Introduction

Obstructive sleep apnea syndrome (OSAS) is a common but often unrecognized disorder [1]. It is characterized by recurrent episodes of intermittent hypoxemia and arousal during sleep, caused by repeated episodes of upper airway collapse [2,3]. Overnight polysomnography (PSG) is considered the gold standard for its diagnosis. There has been increasing recognition that OSAS, due to its hypoxemia, is associated with a higher incidence of many neurological problems, including stroke, cognitive decline, depression, headaches, peripheral neuropathy, and non-arteritic ischemic optic neuropathy (NAION) [4,5,6,7,8].

Glaucoma is an optic neuropathy, in which tissue damage occurs at the level of the optic nerve head, resulting in a characteristic alteration of its appearance, as well as visual field disturbances [9]. Most of these conditions are related to elevated intraocular pressure (IOP), which can also damage the optic nerve directly [10]. However, given that at least some glaucoma patients have low or normal intraocular pressure, other systemic conditions, such as diabetes, cardiovascular disease, and obesity, may also be relevant [11,12].

Whether there is an association between sleep apnea and glaucomatous neuropathy has long been controversial in the literature. Some studies have confirmed the existence of a relationship between sleep apnea and normal-tension glaucoma (NTG), and/or open-angle glaucoma (OAG) [13,14,15,16]. However, other studies have shown no relationship [17,18,19,20]. Thus, we performed this meta-analysis to evaluate whether there is a significant positive association between OSAS and the prevalence of glaucoma.

## Materials and Methods

### Data Sources and Searches

This meta-analysis was carried out according to the PRISMA guidelines [21] (S1 PRISMA Checklist). Two investigators (Yuhua Shi and Kaiming Su) performed a comprehensive literature search, using Embase and PubMed, for related literature published before March 2014. The following terms were used: 1) glaucoma, optic neuropathy, 2) sleep apnea, sleep dysfunction, sleep disordered, OSAS, SAS or OSASHS. Literature language and literature type were not restricted.

We first performed an initial screening of all searched abstracts and subsequently selected the relevant full texts for further investigation. We also searched for “related articles” in PubMed. To find more articles, a manual search of the reference lists of relevant original and review articles was also performed. The two reviewers (Yuhua Shi and Kaiming Su) carried out the initial screening independently; any disagreements were discussed among the authors and resolved by consensus.

### Inclusion and Exclusion Criteria

Any study that met all of the following criteria was included in the quantitative analysis (meta-analysis): (1) the study was of an observational design (cross-sectional, case-control, or cohort studies) and concerned an association between OSAS and glaucoma, (2) clear diagnostic criteria for OSAS were established, (3) diagnostic criteria for glaucoma were also established, (4) OSAS and glaucoma could be used as an outcome in the analysis, and (5) the study included an effective control group.

Studies that met the above criteria with the exception of item 5 (no effective control group) were excluded from the quantitative analysis but included in the review. Case reports and literature reviews were also excluded from the review.

### Data Extraction and Quality Assessment

Information was extracted from all eligible studies by two independent investigators. Differences in the information extracted were discussed among the authors and resolved by consensus. The recorded information for cross-sectional and case-control studies included the name of the first author, publication year, nationality, study design, participant selection, age, type(s) of glaucoma, total numbers of cases and controls, methods for the diagnosis of glaucoma, method used to assess OSAS, adjustment for covariates and the authors’ conclusions. For cohort studies, we also collected the follow-up period.

Because there is no consensus as to the ‘best’ standardized method for assessing the quality of observation studies [22], we designed a five-item scoring scale (each item scoring 0 or 1; 1 being better) [22,23]. The items on the integer scale were representativeness of the cases, whether the assessment of glaucoma was appropriate, whether the assessment of sleep apnea was objective, whether sleep apnea severity was assessed, and controls for confounding. Scores of 0–3 were evaluated as ‘low’ quality while 4 or 5 was considered to indicate ‘high’ quality [22,23]. Two reviewers, Yuhua Shi and Kaiming Su, assessed the quality independently while blinded to each other’s evaluations; disagreements or uncertainties were then settled by discussion.

### Statistical Methods

In this study, the heterogeneity between studies determined the model (a fixed-effects or a random-effects model) used for the meta-analysis. A statistical test for heterogeneity was performed with the I^2^ statistic (values of 25%, 50%, and 75% were considered to represent low, medium, and high degrees of heterogeneity, respectively) and the Q test (heterogeneity was considered statistically significant when P < 0.10). When there was significant heterogeneity (I^2^> 50%), a random-effects model was used to evaluate the pooled OR; otherwise, we used a fixed-effects model.

In cross-sectional and case-control studies, the odds ratio with 95% confidence intervals was used to evaluate the association between OSAS and glaucoma; combined ORs were obtained using the Mantel-Haenszel method. For cohort studies, we calculated the hazard ratio (HR) of glaucoma with the respective 95% CI, if possible. To further evaluate the stability of the results, sensitivity analysis was performed by sequentially excluding each study.

The meta-analysis was performed using Review Manager (ver. 5.2.0). A P value < 0.05 was considered to indicate statistical significance.

## Results

### Literature Search Results and the Characteristics of the Included Studies

**Fig 1:**
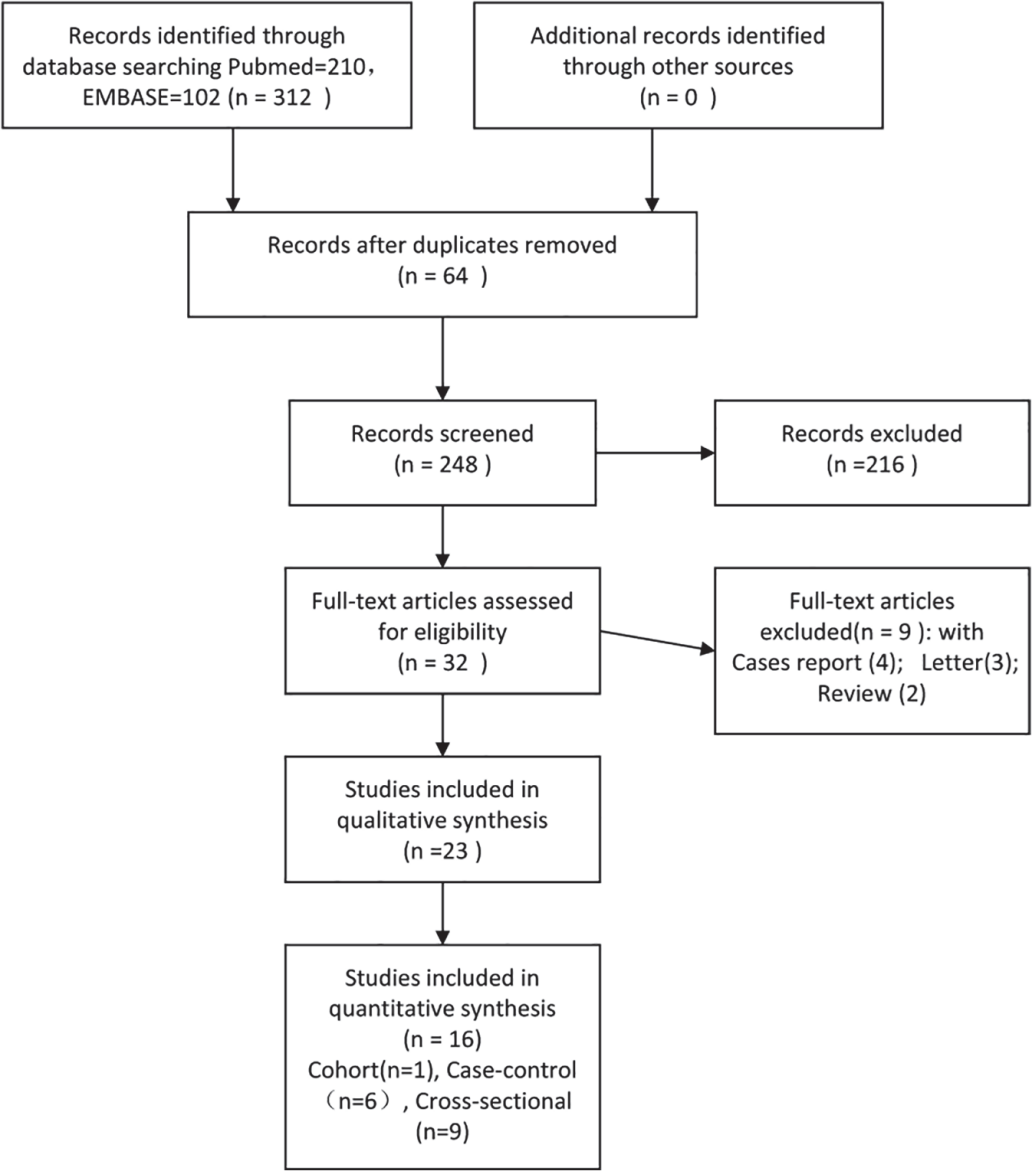
Flow chart of the procedure for identifying studies and the results thereof.

Both investigators agreed on the results of study selection (inclusion/exclusion). The strategies for study identification and study selection are shown in Fig. 1. There were 23 studies focusing on the association between OSAS and glaucoma, but only 16, with a total of 2,278,832 participants, were included in the analysis according to the criteria above (Table 1). These consisted of one retrospective cohort study [14], six case-control studies with OSAS as exposure [15,17,18,19,24,25], and nine cross-sectional studies comparing glaucoma in OSAS and non-OSAS groups [13,16,20,26,27,28,29,30,31] (Table 1). In a study reporting the prevalence of both open-angle glaucoma (OAG) and normal-tension glaucoma (NTG) in sleep apnea patients, data for NTG were excluded because the two sets of data overlapped and could not be merged, while the sample size for OAG was considerably larger than that for NTG [28].

**Table 1:** The 23 observational studies investigating associations between obstructive sleep apnea and glaucoma.

No.	Reference	Nationality& Region	Participants	Mean Age(years)Case/control	Types of Glaucoma	Glaucoma diagnosis	OSAS diagnosis	Glaucoma in OSAS	Glaucoma in control	OSAS in Glaucoma	OSAS in control	Significant Association?	Adjustment for covariates	Scoring	Exclusion reason
**Included cohort Studies**															
1	[14]	Taiwan	Clinical Database	66.7/67.2	OAG	Database record	Database PSG record	114/1012	410/6072			YES	Age, sex, urbanization level, year of index date, hypertension, diabetes, coronary heart disease, hyperlipidemia, obesity, renal disease, migraine, and hypothyroidism	4	/
**Included case-control Studies**															
2	[15]	France	OAG patients and control	>40	OAG	Eye examination,	Sleep questionnaire	/	/	58/212	39/218	YES	/	3	/
3	[24]	USA	NTG patients and control	40–60	NTG	Database record,ICD-9	Sleep history, PSG	/	/	2/37	1/30	YES	/	3	/
4	[17]	USA	OAG patients and control	69/69	OAG	Database record,ICD-9	Database record, ICD-9	/	/	7/667	32/6667	NO	Age, diabetes, lipid metabolism disorders, hypertension, cardiovascular disease, cerebrovascular disease, arterial disease, and migraines	4	/
5	[18]	Australia	OAG patients and control	71/70	OAG	Eye examination	Oximetry monitoring, ODI >20	/	/	9/52	7/60	NO	/	4	/
6	[19]	India	OAG and NTG and control	40–60	OAG,NTG	Eye examination	Sleep history, PSG	/	/	4/40	1/40	NO	/	3	/
7	[25]	Turkey	NTG patients and control	53–78	NTG	Eye examination	PSG, AHI≥20	/	/	10/24	3/24	YES	/	4	/
**Included cross-sectional Studies**															
8	[13]	Italy	Diagnosed OSA, Consecutively with control	74.5/75	NTG	Eye examination	PSG, AHI≥10	3/51	0/40	/	/	YES	/	3	/
9	[31]	Thailand	Suspected OSAS, Consecutively	75/54.7	OAG,NTG	Eye examination	PSG, AHI≥10	6/44	3/42	/	/	NO	/	3	/
10	[26]	China	Suspected OSAS, Consecutively	67.7/72	OAG,NTG	Eye examination	PSG, AHI>5	4/31	0/25	/	/	YES	/	2	/
11	[20]	UK	Suspected OSA, Consecutively	84.3/65.4	OAG,NTG	Eye examination	PSG, ODI≥5	3/89	1/26	/	/	NO	/	3	/
12	[27]	Taiwan	Suspected OSA, Consecutively	76.7/63.1	NTG	Eye examination	PSG, AHI≥5	12/209	0/38	/	/	YES	/	4	/
13	[16]	USA	Medical database	/	Nonspecific	Database record	Database record	228/2725	3410/68235	/	/	YES	/	3	/
14	[28]	USA	Medical database	>40	OAG,	Billing records	Billing records	OAG: 4557/151633; NTG:342/156308	OAG: 50533/2030682; NTG:154330/209916555	/	/	NO	Age, sex, race, region of residence within the US, education level, household net worth, and some medical and ocular conditions including diabetes mellitus, hypertension, obesity, cataract, diabetic retinopathy, and macular degeneration	4	Data of NTG were excluded because of overlapping
15	[30]	Spain	Suspected OSA, Consecutively	79.5/72	OAG,NTG	Eye examination	PSG, AHI≥10	16/202	0/25	/	/	YES	/	3	/
16	[29]	France	Medical database	63.25/61.82	/	Database record	Database record	240/6754	89/2826	/	/	NO	Age, sex, height, weight, body mass index, arterial hypertension, tobacco consumption, high cholesterol levels, high triglyceride levels, and thyroid dysfunction.	4	/
**Excluded Studies**															
17	[32]	Switzerland	Diagnosed Glaucoma patients	39–81	NTG	Eye examination	PSG, RDI >10	/	/	7/16	/	YES	/	/	No effective control
18	[33]	France	Diagnosed Glaucoma patients	57.85	OAG NTG	Eye examination	Sleep history, PSG	/	/	15/31	/	YES	/	/	No effective control
19	[34]	Switzerland	OSAS, Consecutively	88.4	OAG,NTG	Eye examination	PSG, RDI>10	5 /69	/	/	/	YES	/	/	No effective control
20	[35]	Israel	Diagnosed OSAS, Consecutively	83.3	OAG	Eye examination	PSG, RDI>10	5/228	/	/	/	NO	/	/	No effective control
21	[36]	Hong Kong	Diagnosed OSAS, Consecutively	86.11/86.66	/	Eye examination	PSG, AHI≥20	3/82(eyes)	2/68(eyes)	/	/	YES	/	/	Not enough data
22	[37]	USA	Suspected OSA	65	OAG NTG	Eye examination	PSG, AHI>15	27/100	/	/	/	YES	/	/	No effective control
23	[38]	Iran	Diagnosed OSAS	51.77	OAG	Eye examination	PSG, AHI>5	9/90	/	/	/	YES	/	/	No effective control

OSAS, obstructive sleep apnea syndrome; OAG, open-angle glaucoma; NTG, normal-tension glaucoma; ICD-9, International Classification of Diseases, 9th edition

PSG, polysomnography; ODI, oxygen desaturation index; AHI, apnea hypopnea index.

The remaining seven studies [32,33,34,35,36,37,38] were excluded. One study had insufficient data for extraction; it reported the number of eyes with glaucoma but not the number of patients [36]. The other six were excluded because they included no effective control group; those articles concluded with or without a significant association between glaucoma and OSAS were based only on a high or low prevalence of glaucoma (or OSAS) in patients with OSAS (or glaucoma). For example, one article reported that the prevalence of glaucoma in patients with sleep apnea was 7.2%, which is higher than the 2% expected in the general population [34].

Regarding the diagnostic methods for glaucoma, the included reports used two criteria; 10 used systematic eye examinations, including visual field, optic nerve head cupping, anterior chamber angle, and intraocular pressure [13,15,18,19,20,25,26,27,30,31], while 6 were based on database or billing records [14,16,17,24,28,29]. Regarding the diagnostic criteria for OSAS, nine used objective evaluations, such as PSG and oximetry monitoring [13,18,19,20,25,26,27,30,31], five were based on database or billing records [14,16,17,24,29], and one was based only on a sleep questionnaire [15].

The study population and data source were from a single hospital in 12 studies (6 case-control studies and 6 cross-sectional studies). The patients in five case-control studies were diagnosed glaucoma patients at an eye clinic [15,18,19,24,25]; the other case-control study was from a hospital-based database [17]. In contrast, all patients in the cross-sectional studies with suspected OSAS were from sleep centers [13,20,26,27,30,31]. Data for the four other included studies were from a database that contained information on patients from several hospitals.

Two of the six case-control studies matched by age or gender [17,25], three case-control studies reported no difference between cases and controls in age, gender, and/or body mass index (BMI) in the design [15,18,24]; two cross-sectional studies [27,29] reported associations between OSAS and glaucoma according to OSAS severity. The quality score for the cohort study was 4. The mean quality scores of the case-control studies and cross-sectional studies were 3.5 (range, 2–4) and 3.2 (range, 2–4), respectively. A detailed assessment of the quality of the included studies is provided in S1 Table.

### Pooled-analysis Results

**Cohort studies.** Because only one cohort study was included, a meta-analysis was not performed for this series. The population-based retrospective cohort study included 1012 OSAS patients and 6072 controls, aged 40 years or older. After adjusting for several confounders, such as monthly income, place of residence, diabetes, cardiovascular disease, and obesity, the hazard ratio for OAG within the 5-year period for subjects with OSAS was 1.67 (95% CI = 1.30–2.17; P<0.001) [14].

**Fig 2:**
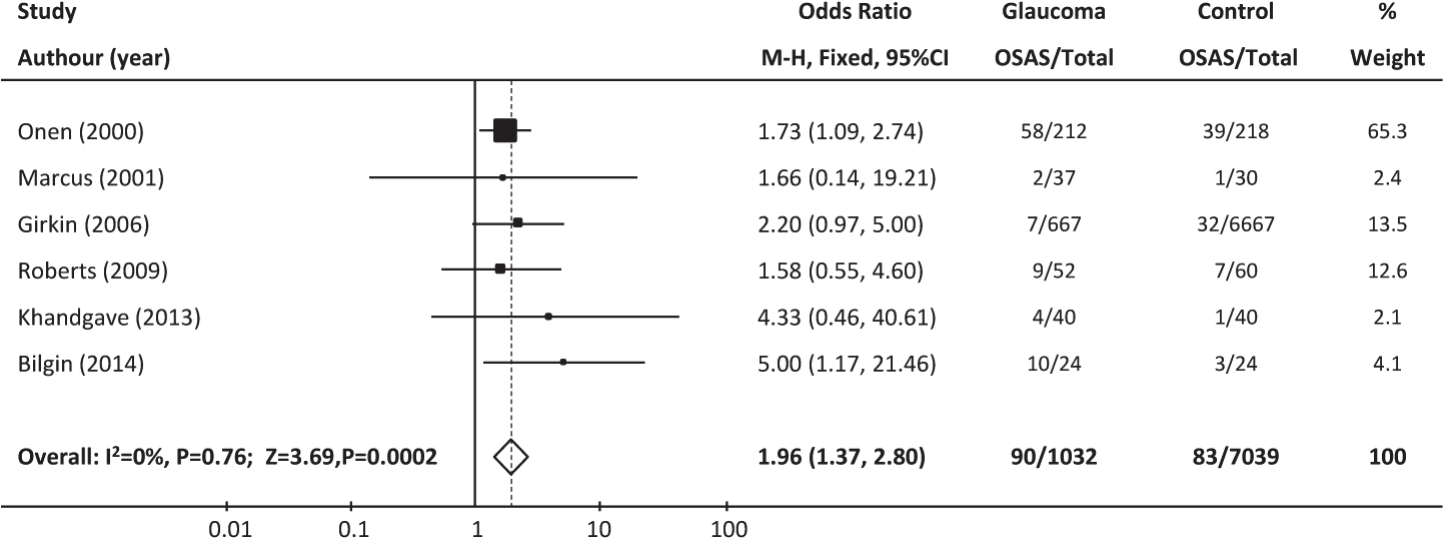
Forest plot of case-control studies showing the odds ratios (ORs) with 95% confidence intervals (95% CI) of OSAS for participants with and without glaucoma. The squares and horizontal lines represent the study-specific ORs and 95% CIs. The sizes of the squares reflect the statistical weights of the studies. The pooled OR is indicated by a diamond (fixed-effect model).

**Case-control studies.** Of the six case-control studies, which involved 1032 glaucoma patients and 7039 controls, two showed positive associations between OSAS and glaucoma, while four showed no association. Thus, we performed a pooled analysis to assess the overall results. A fixed-effects model was used for the pooled analysis because no statistical heterogeneity was found among the studies (χ^2^ = 2.60, I^2^ = 0%, P = 0.76). The results showed a significant relationship between glaucoma and OSAS (pooled OR = 1.96, 95%CI = 1.37–2.80; P = 0.0002; Fig. 2).

Sensitivity analysis: The result of the heterogeneity test and the significance of the pooled OR were not greatly influenced by the omission of a single study (Onen 2000, weight: 65.3%) in which the OSAS diagnosis was based only on a questionnaire (χ^2^ = 1.96, I^2^ = 0%; P = 0.74; pooled OR = 2.40, 95% CI = 1.38–4.18, P = 0.002). We also evaluated the pooled OR for the remaining studies by excluding each study sequentially, which produced a range from 1.83 (95% CI = 1.17–2.65; P = 0.001) to 2.40 (95% CI = 1.38–4.18; P = 0.002), also indicating a ‘stable’ result for the association.

Subgroup analysis: Subjects in the studies were from various geographical regions, and racial differences have been reported in patients with OSAS. Thus, a subgroup analysis of the case control studies according to region was also conducted. The pooled ORs of the studies from United States + Europe [15,17,24] and other countries [18,19,25] were 1.80 (95% CI = 1.21–2.69, P = 0.004) and 2.64 (95% CI = 1.21–5.77, P = 0.02), respectively. Thus, both comparisons showed a positive association between OSAS and glaucoma.

**Fig 3:**
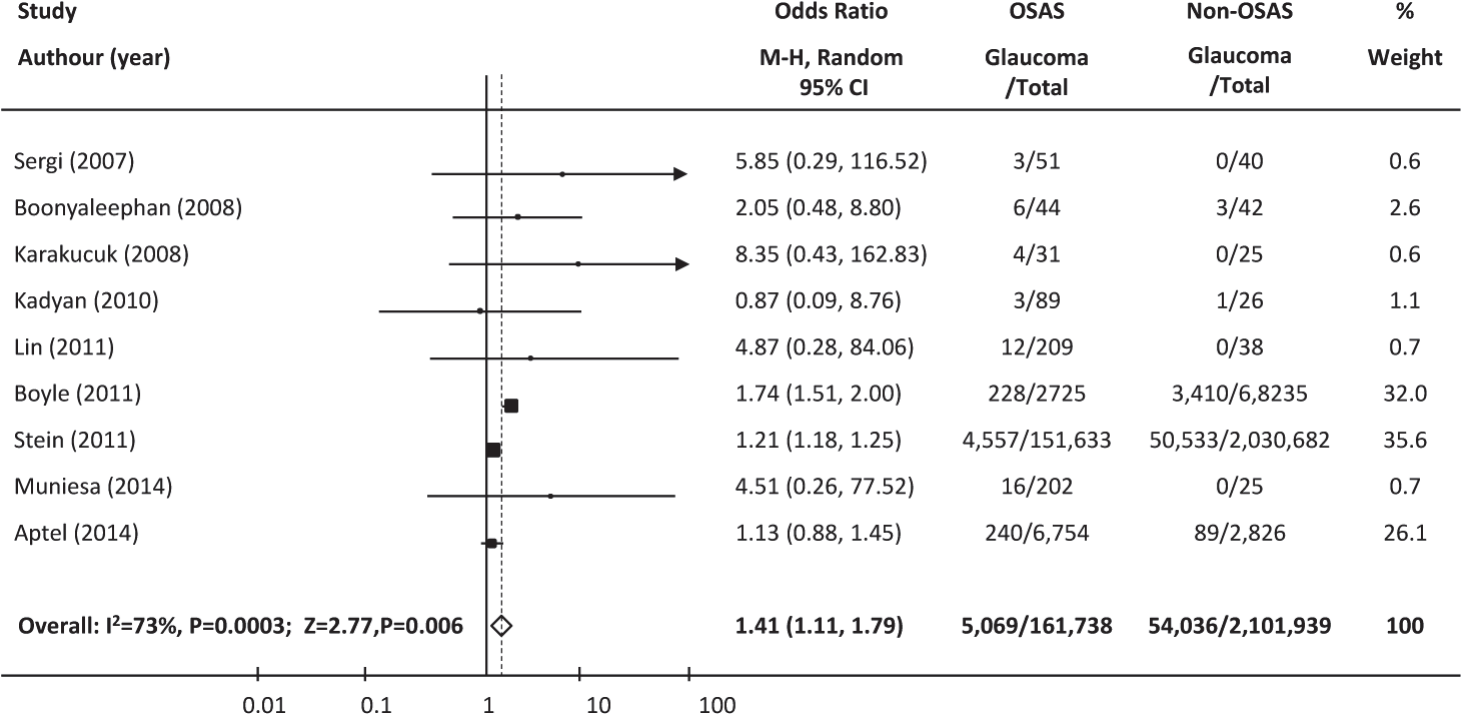
Forest plot of cross-sectional studies showing the odds ratios (ORs) with 95% confidence intervals (95% CI) of glaucoma for participants with and without OSAS. **The squares and horizontal lines represent the study-specific ORs and 95% CIs.** The sizes of the squares reflect the statistical weights of the studies. The pooled OR is indicated by a diamond (random-effect model).

**Cross-sectional studies.** In nine cross-sectional studies, glaucoma was present in 5,069 of 161,738 patients with OSAS and in 54,036 of 2,101,939 patients without OSAS. Because the heterogeneity test showed medium-to-high heterogeneity among the individual studies (heterogeneity test χ^2^ = 29.26, I^2^ = 73%, P = 0.0003), a pooled analysis was performed using a random-effects model. The overall pooled OR for glaucoma was 1.41 (95% CI = 1.11–1.79, P = 0.006) in the OSAS group versus the non-OSAS group (Fig. 3).

Sensitivity analysis: To explore the potential sources of heterogeneity observed in this analysis, we excluded each study sequentially to determine its effect on the main summary estimate. The pooled OR ranged from 1.21(95%CI = 1.18–1.28, P<0.0001) to 1.55 (95% CI = 1.12–2.13, P = 0.007). After removing the study of Boyle 2011 [16], which enrolled patients from a veterans’ electronic medical database, the variation across the studies disappeared and the I^2^ statistic dropped from 73% to 0% (heterogeneity test χ^2^ = 5.29, P = 0.62). This study suggested that the diagnosis rate of glaucoma was 8.4% in patients with sleep apnea and 5% in those without, markedly higher than in the two other large-sample studies. The pooled OR of glaucoma in the remaining eight studies was 1.28 (95% CI = 1.18–1.25, P<0.00001).

**Fig 4:**
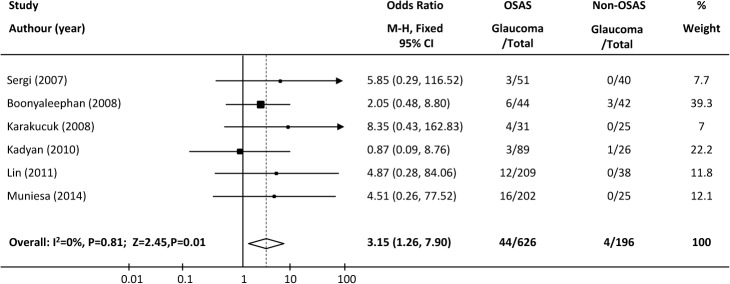
Forest plot of six cross-sectional studies with small sample sizes showing the odds ratios (ORs) with 95% confidence intervals (95% CI) of glaucoma for participants with and without OSAS. The squares and horizontal lines represent the study-specific ORs and 95% CIs. The sizes of the squares reflect the statistical weights of the studies. The pooled OR is indicated by a diamond (fixed-effect model).

Subgroup analysis: Because the diagnosis of OSAS in each of the three large-sample-size studies [16,28,29] was based on billing codes or medical records, not polysomnography, a subgroup analysis was performed of the six smaller-sample-size studies [13,20,26,27,30,31], which consequently had wider confidence intervals and in which all diagnoses of OSAS were based on objective measurements. The pooled analysis of these studies also demonstrated a significant association between OSAS and glaucoma (χ^2^ = 2.25, I^2^ = 0, p = 0.81; pooled OR = 3.15, 95%CI = 1.26–7.90, P = 0.01; Fig. 4).

A subgroup analysis of the cross-sectional studies according to region was also conducted. The pooled ORs of the studies from the United States/Europe [13,16,20,28,29,30] and Asian countries [26,27,31] in the cross-sectional studies were 1.36 (95% CI = 1.06–1.75, P = 0.1; τ^2^ = 26.31, I^2^ = 81%; P <0.0001) and 3.39 (95% CI = 1.06–10.81, P = 0.04; χ^2^ = 0.87, I^2^ = 0%; P = 0.65), respectively. Thus, both showed a positive association between OSAS and glaucoma.

### Confounding factors

Only four of the studies reported their results after adjusting for confounding factors. One was the cohort study [14] that showed a significant association, as described above. The second was the case-control study of Girkin et al. [17]. After adjusting for some systemic diseases, such as diabetes, lipid metabolism disorders, hypertension, and cardiovascular disease, a conditional logistic regression analysis showed no significant relationship between glaucoma and sleep apnea (unadjusted OR, 2.20; 95% CI, 0.967–5.004; P = 0.06; adjusted OR, 1.80; 95% CI, 0.76–4.23; P = 0.18). The two other studies were of cross-sectional design [28,29]. Both showed no significant association between the two conditions after adjusting for several confounding systemic diseases. The reported adjusted ORs were 1.01 (95% CI, 0.98–1.05) [28] and 1.13 (95% CI, 0.87–1.47) [29], respectively.

Due to the limited number of studies involved and their marked heterogeneity (one cohort, one case-control, two cross-sectional), it was inappropriate to combine their data. Thus, no meta-regression was performed.

Pooled data from all 16 observational studies were also not analyzed, mainly because the subjects in the cross-sectional and cohort studies were usually suspected OSAS patients in sleep centers or a large medical database, while patients in the case-control studies were usually from an eye clinic. The biases in patient selection in those studies resulted inconsiderable clinical variability and heterogeneity, making further statistical analyses difficult.

It should be emphasized that the reported pooled estimates for the case-control studies and cross-sectional studies were based on unadjusted ORs, and the findings from studies that adjusted for potential confounders were inconsistent.

## Discussion

Determining an association between OSAS and glaucoma could be meaningful from a public health point of view, given that both are common medical disorders. This meta-analysis of six case-control and nine cross-sectional studies confirmed a statistically significant association between OSAS and glaucoma, consistent with a previously reported retrospective cohort study. Given that no prospective study was included, and the pooled results in this study represent unadjusted estimates, which are likely to be confounded by other risk factors, these results should be confirmed in further studies.

Our analysis included five studies based on patient databases [14,16,17,28,29], in which the sample sizes were very large, and the diagnosis of sleep apnea was from billing records, sleep history, or disease classification diagnosis codes, but not polysomnography. Because glaucoma directly affects vision, whereas symptoms of sleep apnea may not be as obvious and are prone to be neglected by potential patients, the rate of undiagnosed sleep apnea in a database of patients should be much higher than that of glaucoma, which may introduce selection bias. However, because of their very large sample sizes, these studies were included in the analysis. Additionally, to reduce the interference, we also performed subgroup analyses of those studies with an objective OSAS diagnosis, but with smaller sample size and, consequently, wider confidence intervals. A significant association between OSAS and glaucoma was also found in these results.

It has been reported that the clinical characteristics of patients with OSAS differ according to ethnicity [39]. For example, past studies have found that the relationship between hypertension and symptoms of OSAS may be mediated by ethnicity and race. In this study, subgroup analyses showed that the significant association between OSAS and glaucoma was unaffected by the study region. However, due to the limited number of included studies, whether these results are applicable to all races/ethnicities should be investigated further.

A meta-analysis including observational studies cannot ‘prove’ causation. However, two theories, a vascular theory and a mechanical theory, can explain the potential association between OSAS and glaucoma [14,40]. The vascular theory postulates that the upper airway collapse during sleep in OSAS patients would lead to repeated or prolonged episodes of hypoxia, which may reduce the oxygen supply to the optic nerve, and subsequently lead to optic neuropathy. The mechanical theory considers that OSAS has the potential to raise the intraocular pressure through changes in sleep architecture and through an increase in sympathetic tone; this raised intraocular pressure will lead to axon damage at the optic nerve, and eventually cause glaucoma. Other theories, including inflammation, oxidative stress, and hypercapnia, have also been suggested to explain the association between glaucoma and sleep apnea [41,42].

Because of the limited number of related studies, the issue of confounding could not be examined by meta-regression in this study. A meta-analysis should be carried out using ‘raw’ data, and thus the pooled odds ratios represent unadjusted estimates, which are likely to be heavily confounded by other risk factors such as age, gender, and hypertension. While the apparent association between OSAS and glaucoma may be of interest in its own right, any attempt to explore this relationship further would seem to be incomplete without examining the roles of potential confounders. In fact, patients with OSAS have significantly higher risks of *all* comorbidities considered [5–7]. Thus, OSAS may simply be a marker of poor (vascular, obesity) health, and not necessarily an independent risk factor for glaucoma.

Two other limitations of this meta-analysis should also be considered. First, none of the studies selected their participants randomly; most were based on patients attending a clinic, Thus, selection bias may exist. Second, due to the limited number of studies involved relating to OSAS and glaucoma, only two studies included information on the severity of OSAS. Thus, we were unable to evaluate whether patients with more severe OSAS had a higher odds ratio for glaucoma than those with milder OSAS.

In recent years, OSAS has been increasingly recognized as a risk factor for both systemic and ocular diseases. For example, past studies have demonstrated that the risks of developing diabetes, stroke, hypertension, and atherosclerosis are increased in OSAS patients [5,43,44]. In ophthalmology, besides glaucoma, papill edema, non-arteritic ischemic optic neuropathy (NAION), and other ocular disorders have also been considered as potential lesions due to intermittent nocturnal hypoxia caused by OSAS [4,45,46]. Based on previous results, combined with this study, it is important for ophthalmologists, otolaryngologists, and sleep physicians to better understand this relationship, so that both relevant ocular disorders and sleep apnea can be recognized and diagnosed and treated early.

## Conclusions

In conclusion, this is the first reported meta-analysis with the aim of understanding the association between OSAS and glaucoma. This meta-analysis of six case-control and nine cross-sectional studies confirmed a statistically significant association between OSAS and glaucoma, consistent with a previous retrospective cohort study. Prospective cohort studies that take into consideration potential confounders, or include data from interventional trials to determine whether a reduction in OSAS status is associated with a reduced incidence of glaucoma are needed to clarify whether OSAS is an independent risk factor for glaucoma.

## Supporting Information

S1 PRISMA Checklist PRISMA Checklist of this meta-analysis. (DOC)

S1 Table The 23 observational studies investigating associations between obstructive sleep apnea and glaucoma. (DOC)
