# Supplementary material for: Association between Glaucoma and Obstructive Sleep Apnea Syndrome: A Meta-Analysis and Systematic Review
Source: PLoS One. 2015 Feb 23;10(2):e0115625. doi: 10.1371/journal.pone.0115625 (PMC4338237; doi:10.1371/journal.pone.0115625)
Supplement: S1 Table — (DOC) [file pone.0115625.s002.doc]

**Table 1. The 23 observational studies investigating associations between obstructive sleep apnea and glaucoma.**

| **No.** | **Reference** | **Nationality & Region** | **Participants** | **Mean Age (years) Case/control** | **Types of Glaucoma** | **Glaucoma diagnosis** | **OSAS diagnosis** | **Glaucoma in OSAS** | **Glaucoma in control** | **OSAS in Glaucoma** | **OSAS in control** | **Significant Association?** | **Adjustment for covariates** | **Scoring** | **Exclusion reason** |
| --- | --- | --- | --- | --- | --- | --- | --- | --- | --- | --- | --- | --- | --- | --- | --- |
| **Included cohort Studies** | | | | | | | | | | | | | | | |
| 1 | (L*in et a*l., 2013) | Taiwan | Clinical Database | 66.7/67.2 | OAG | Database record | Database PSG record | 114/1012 | 410/6072 |  |  | YES | Age, sex, urbanization level, year of index date, hypertension, diabetes, coronary heart disease, hyperlipidemia, obesity, renal disease,　migraine, and hypothyroidism | 4 | / |
| **Included case-control Studies** | | | | | | | | | | | | | | | |
| 2 | (On*en et a*l., 2000) | France | OAG patients and control | >40 | OAG | Eye examination, | Sleep questionnaire | / | / | 58/212 | 39/218 | YES | / | 3 | / |
| 3 | (Marc*us et a*l., 2001) | USA | NTG patients and control | 40-60 | NTG | Database record, ICD-9 | Sleep history, PSG | / | / | 2/37 | 1/30 | YES | / | 3 | / |
| 4 | (Girk*in et a*l., 2006) | USA | OAG patients and control | 69/69 | OAG | Database record, ICD-9 | Database record, ICD-9 | / | / | 7/667 | 32/6667 | NO | Age, diabetes, lipid metabolism disorders, hypertension, cardiovascular disease, cerebrovascular　disease, arterial disease, and migraines | 4 | / |
| 5 | (Rober*ts et a*l., 2009) | Australia | OAG patients and control | 71/70 | OAG | Eye examination | Oximetry monitoring, ODI >20 | / | / | 9/52 | 7/60 | NO | / | 4 | / |
| 6 | (Khandga*ve et a*l., 2013) | India | OAG and NTG and control | 40-60 | OAG,NTG | Eye examination | Sleep history, PSG | / | / | 4/40 | 1/40 | NO | / | 3 | / |
| 7 | (Bilgin, 2014) | Turkey | NTG patients and control | 53-78 | NTG | Eye examination | PSG, AHI≥20 | / | / | 10/24 | 3/24 | YES | / | 4 | / |
| **Included cross-sectional Studies** | | | | | | | | | | | | | | | |
| 8 | (Ser*gi et a*l., 2007) | Italy | Diagnosed OSA, Consecutively with control | 74.5/75 | NTG | Eye examination | PSG, AHI≥10 | 3/51 | 0/40 | / | / | YES | / | 3 | / |
| 9 | (Boonyaleephan. and Neruntarat., 2008) | Thailand | Suspected OSAS, Consecutively | 75/54.7 | OAG,NTG | Eye examination | PSG, AHI≥10 | 6/44 | 3/42 | / | / | NO | / | 3 | / |
| 10 | (Karakuc*uk et a*l., 2008) | China | Suspected OSAS, Consecutively | 67.7/72 | OAG,NTG | Eye examination | PSG, AHI>5 | 4/31 | 0/25 | / | / | YES | / | 2 | / |
| 11 | .(Kady*an et a*l., 2010) | UK | Suspected OSA, Consecutively | 84.3/65.4 | OAG,NTG | Eye examination | PSG, ODI≥5 | 3/89 | 1/26 | / | / | NO | / | 3 | / |
| 12 | (L*in et a*l., 2011) | Taiwan | Suspected OSA, Consecutively | 76.7/63.1 | NTG | Eye examination | PSG, AHI≥5 | 12/209 | 0/38 | / | / | YES | / | 4 | / |
| 13 | (Boyle-Walk*er et a*l., 2011) | USA | Medical database | / | Nonspecific | Database record | Database record | 228/2725 | 3410/68235 | / | / | YES | / | 3 | / |
| 14 | (Ste*in et a*l., 2011) | USA | Medical database | >40 | OAG, | Billing records | Billing records | OAG: 4557/151633; NTG:342/156308 | OAG: 50533/2030682; NTG:154330/209916555 | / | / | NO | Age, sex, race, region of residence within the US, education level, household net worth, and some medical and ocular conditions including diabetes mellitus, hypertension, obesity, cataract, diabetic retinopathy, and macular degeneration | 4 | Data of NTG were excluded because of overlapping |
| 15 | (Munie*sa et a*l., 2014) | Spain | Suspected OSA, Consecutively | 79.5/72 | OAG,NTG | Eye examination | PSG, AHI≥10 | 16/202 | 0/25 | / | / | YES | / | 3 | / |
| 16 | (Apt*el et a*l., 2014) | France | Medical database | 63.25/61.82 | / | Database record | Database record | 240/6754 | 89/2826 | / | / | NO | Age, sex, height, weight, body mass index, arterial hypertension, tobacco consumption, high cholesterol levels, high triglyceride levels, and thyroid dysfunction. | 4 | / |
| **Excluded Studies** | | | | | | | | | | | | | | | |
| 17 | (Moj*on et a*l., 2002) | Switzerland | Diagnosed Glaucoma patients | 39-81 | NTG | Eye examination | PSG, RDI >10 | / | / | 7/16 | / | YES | / | / | No effective control |
| 18 | (Blumen Oha*na et a*l., 2010) | France | Diagnosed Glaucoma patients | 57.85 | OAG NTG | Eye examination | Sleep history, PSG | / | / | 15/31 | / | YES | / | / | No effective control |
| 19 | (Moj*on et a*l., 1999) | Switzerland | OSAS, Consecutively | 88.4 | OAG，NTG | Eye examination | PSG, RDI>10 | 5 /69 | / | / | / | YES | / | / | No effective control |
| 20 | (Gey*er et a*l., 2003) | Israel | Diagnosed OSAS, Consecutively | 83.3 | OAG | Eye examination | PSG, RDI>10 | 5/228 | / | / | / | NO | / | / | No effective control |
| 21 | (Tsa*ng et a*l., 2006) | Hong Kong | Diagnosed OSAS, Consecutively | 86.11/86.66 | / | Eye examination | PSG, AHI≥20 | 3/82(eyes) | 2/68(eyes) | / | / | YES | / | / | Not enough data |
| 22 | (Bend*el et a*l., 2008) | USA | Suspected OSA | 65 | OAG NTG | Eye examination | PSG, AHI>15 | 27/100 | / | / | / | YES | / | / | No effective control |
| 23 | (Manavi*at et a*l., 2014) | Iran | Diagnosed OSAS | 51.77 | OAG | Eye examination | PSG, AHI>5 | 9/90 | / | / | / | YES | / | / | No effective control |

OSAS, obstructive sleep apnea syndrome; OAG, open-angle glaucoma; NTG, normal-tension glaucoma; ICD-9, International Classification of Diseases, 9th edition; PSG, polysomnography; ODI, oxygen desaturation index; AHI, apnea hypopnea index.

Aptel, F., Chiquet, C., Tamisier, R., Sapene, M., Martin, F., Stach, B., Grillet, Y., Levy, P. &Pepin, J. L. (2014). Association between glaucoma and sleep apnea in a large French multicenter prospective cohort. *Sleep Med*.

Bendel, R. E., Kaplan, J., Heckman, M., Fredrickson, P. A. &Lin, S. C. (2008). Prevalence of glaucoma in patients with obstructive sleep apnoea--a cross-sectional case-series. *Eye (Lond)* 22(9): 1105-1109.

Bilgin, G. (2014). Normal-tension glaucoma and obstructive sleep apnea syndrome: a prospective study. *BMC Ophthalmol* 14: 27.

Blumen Ohana, E., Blumen, M. B., Bluwol, E., Derri, M., Chabolle, F. &Nordmann, J. P. (2010). Primary open angle glaucoma and snoring: prevalence of OSAS. *Eur Ann Otorhinolaryngol Head Neck Dis* 127(5): 159-164.

Boonyaleephan., S. &Neruntarat., C. (2008). The association of primary open-angle glaucoma / normal tension glaucoma and obstructive sleep apnea in Thai patients. *Journal of Medicine and Health Sciences* 15(1): 87-94.

Boyle-Walker, M., Semes, L. P., Clay, O. J., Liu, L. &Fuhr, P. (2011). Sleep apnea syndrome represents a risk for glaucoma in a veterans' affairs population. *ISRN Ophthalmol* 2011: 920767.

Geyer, O., Cohen, N., Segev, E., Rath, E. Z., Melamud, L., Peled, R. &Lavie, P. (2003). The prevalence of glaucoma in patients with sleep apnea syndrome: same as in the general population. *Am J Ophthalmol* 136(6): 1093-1096.

Girkin, C. A., McGwin, G., Jr., McNeal, S. F. &Owsley, C. (2006). Is there an association between pre-existing sleep apnoea and the development of glaucoma? *Br J Ophthalmol* 90(6): 679-681.

Kadyan, A., Asghar, J., Dowson, L. &Sandramouli, S. (2010). Ocular findings in sleep apnoea patients using continuous positive airway pressure. *Eye (Lond)* 24(5): 843-850.

Karakucuk, S., Goktas, S., Aksu, M., Erdogan, N., Demirci, S., Oner, A., Arda, H. &Gumus, K. (2008). Z：Ocular blood flow in patients with obstructive sleep apnea syndrome (OSAS). *Graefes Arch Clin Exp Ophthalmol* 246(1): 129-134.

Khandgave, T. P., Puthran, N., Ingole, A. B. &Nicholson, A. D. (2013). The assessment of sleep apnoea as a risk factor in glaucoma. *J Clin Diagn Res* 7(7): 1391-1393.

Lin, C. C., Hu, C. C., Ho, J. D., Chiu, H. W. &Lin, H. C. (2013). Obstructive sleep apnea and increased risk of glaucoma: a population-based matched-cohort study. *Ophthalmology* 120(8): 1559-1564.

Lin, P. W., Friedman, M., Lin, H. C., Chang, H. W., Wilson, M. &Lin, M. C. (2011). Normal tension glaucoma in patients with obstructive sleep apnea/hypopnea syndrome. *J Glaucoma* 20(9): 553-558.

Manaviat, M. R., Besharati, M. R., Azarpeikan, A. R. &Halvani, A. (2014). Effect of sleep therapy on intraocular pressure. *Zahedan J Res Med Sci* 16(1): 44-47.

Marcus, D. M., Costarides, A. P., Gokhale, P., Papastergiou, G., Miller, J. J., Johnson, M. H. &Chaudhary, B. A. (2001). Sleep disorders: a risk factor for normal-tension glaucoma? *J Glaucoma* 10(3): 177-183.

Mojon, D. S., Hess, C. W., Goldblum, D., Boehnke, M., Koerner, F., Gugger, M., Bassetti, C. &Mathis, J. (2002). Normal-tension glaucoma is associated with sleep apnea syndrome. *Ophthalmologica* 216(3): 180-184.

Mojon, D. S., Hess, C. W., Goldblum, D., Fleischhauer, J., Koerner, F., Bassetti, C. &Mathis, J. (1999). High prevalence of glaucoma in patients with sleep apnea syndrome. *Ophthalmology* 106(5): 1009-1012.

Muniesa, M., Sanchez-de-la-Torre, M., Huerva, V., Lumbierres, M. &Barbe, F. (2014). Floppy eyelid syndrome as an indicator of the presence of glaucoma in patients with obstructive sleep apnea. *J Glaucoma* 23(1): e81-85.

Onen, S. H., Mouriaux, F., Berramdane, L., Dascotte, J. C., Kulik, J. F. &Rouland, J. F. (2000). High prevalence of sleep-disordered breathing in patients with primary open-angle glaucoma. *Acta Ophthalmol Scand* 78(6): 638-641.

Roberts, T. V., Hodge, C., Graham, S. L., Burlutsky, G. &Mitchell, P. (2009). Prevalence of nocturnal oxygen desaturation and self-reported sleep-disordered breathing in glaucoma. *J Glaucoma* 18(2): 114-118.

Sergi, M., Salerno, D. E., Rizzi, M., Blini, M., Andreoli, A., Messenio, D., Pecis, M. &Bertoni, G. (2007). Prevalence of normal tension glaucoma in obstructive sleep apnea syndrome patients. *J Glaucoma* 16(1): 42-46.

Stein, J. D., Kim, D. S., Mundy, K. M., Talwar, N., Nan, B., Chervin, R. D. &Musch, D. C. (2011). The association between glaucomatous and other causes of optic neuropathy and sleep apnea. *Am J Ophthalmol* 152(6): 989-998 e983.

Tsang, C. S., Chong, S. L., Ho, C. K. &Li, M. F. (2006). Moderate to severe obstructive sleep apnoea patients is associated with a higher incidence of visual field defect. *Eye (Lond)* 20(1): 38-42.
